# Amino Acid Metabolism in Liver Mitochondria: From Homeostasis to Disease

**DOI:** 10.3390/metabo15070446

**Published:** 2025-07-02

**Authors:** Ranya Erdal, Kıvanç Birsoy, Gokhan Unlu

**Affiliations:** 1 Laboratory of Metabolic Regulation and Genetics, The Rockefeller University, New York, NY 10065, USA; ranyaerdal@gmail.com; 2 Medical Scientist Training Program, Hacettepe University Faculty of Medicine, Ankara 06230, Turkey

**Keywords:** liver, mitochondria, amino acids, metabolism, inborn errors of metabolism, cancer

## Abstract

Hepatic mitochondria play critical roles in sustaining systemic nutrient balance, nitrogen detoxification, and cellular bioenergetics. These functions depend on tightly regulated mitochondrial processes, including amino acid catabolism, ammonia clearance via the urea cycle, and transport through specialized solute carriers. Genetic disruptions in these pathways underlie a range of inborn errors of metabolism, often resulting in systemic toxicity and neurological dysfunction. Here, we review the physiological functions of hepatic mitochondrial amino acid metabolism, with a focus on subcellular compartmentalization, disease mechanisms, and therapeutic strategies. We discuss how emerging genetic and metabolic interventions—including dietary modulation, cofactor replacement, and gene therapy—are reshaping treatment of liver-based metabolic disorders. Understanding these pathways offers mechanistic insights into metabolic homeostasis and reveals actionable vulnerabilities in metabolic disease and cancer.

## 1. Introduction

The liver is the principal organ responsible for orchestrating nutrient availability by coordinating hormonal, neural, and nutritional inputs. This role depends on the metabolic plasticity of hepatocytes and their ability to adapt to diverse physiological demands [1]. At the tissue level, hepatic function is organized along a portal–central axis that defines metabolic zonation—a spatial blueprint that partitions key biochemical activities. Periportal hepatocytes are enriched for amino acid uptake, catabolism, and ureagenesis, while perivenous cells preferentially express glutamine synthetase to recycle residual ammonia [2]. This division of labor enables efficient nitrogen handling and contributes to systemic pH balance. Within individual hepatocytes, mitochondria occupy a central position in amino acid metabolism. They house key enzymatic reactions for nitrogen disposal, carbon backbone oxidation, and biosynthetic precursor generation. Unlike carbohydrates and lipids, which have dedicated storage forms, amino acids are largely protein-incorporated and lack long-term reservoirs. Their balance is therefore regulated through diet, proteolysis, and mitochondrial degradation. The liver processes these inputs via tightly regulated pathways that feed into the TCA cycle, urea production, and other downstream fates.

Disruptions in mitochondrial amino acid metabolism are associated with a spectrum of inherited metabolic disorders. These include defects in glycine cleavage, branched-chain amino acid oxidation, and urea cycle function—each associated with systemic toxicity and characteristic biochemical signatures. Neurological symptoms are frequent, reflecting the brain’s sensitivity to circulating metabolic byproducts. In parallel, growing evidence shows that cancer cells hijack these same mitochondrial programs to fuel anabolic growth and redox balance, often through rewiring of enzymes such as GLS2, GLUD1, or CPS1. In this review, we explore hepatic mitochondrial amino acid metabolism with a focus on spatial organization, genetic disruption, and therapeutic modulation. We highlight key enzymatic steps and transporter dependencies and examine how new approaches—from cofactor replacement to gene therapy—are reshaping treatment paradigms for liver-based metabolic diseases. Finally, we discuss how tumors exploit mitochondrial circuits and whether this knowledge may yield new therapeutic approaches.

## 2. Mitochondrial Amino Acid Catabolism: Pathways and Disorders

The metabolic fate of amino acids within the liver is predominantly influenced by the overall metabolic state of the organism. Although glycolysis and fatty acid oxidation are the primary pathways for energy production, the catabolism of amino acids can contribute approximately 10–15% of the total energy yield [3]. The degradation of amino acids yields carbon skeletons and amino groups that are redirected into core metabolic pathways. Carbon backbones are typically converted into α-keto acids, which can feed directly into the tricarboxylic acid (TCA) cycle within mitochondria, linking amino acid catabolism to energy production and biosynthesis of proteins. The removal of amino groups from amino acids occurs through deamination reactions, producing ammonia, which in turn is either incorporated into biosynthetic pathways for the synthesis of nitrogenous compounds or converted to urea through the urea cycle for subsequent excretion. Defects in mitochondrial amino acid catabolism can disrupt hepatic function and lead to systemic metabolic toxicity. Because many toxic intermediates cross the blood–brain barrier, these disorders frequently affect the central nervous system. Organic acidemias, for example, result from defective metabolism of branched-chain or other amino acids and are associated with the accumulation of α-keto acids that impair brain function. Therapeutic strategies typically center on dietary protein restriction, cofactor supplementation, and management of acute metabolic crises.

### 2.1. Glycine Degradation

Glycine is metabolized by a mitochondrial multienzyme complex known as the glycine cleavage system (GCS). This system comprises four distinct proteins: P-Protein (glycine carboxylase, encoded by the *GLDC* gene), H-Protein (hydrogen carrier protein, encoded by the *GCSH* gene), T-Protein (amino methyltransferase, encoded by the *AMT* gene), and L-Protein (dihydrolipoamide dehydrogenase, encoded by the *DLD* gene). Nonketotic hyperglycinemia (NKH) is an autosomal recessive disorder arising from genetic mutations within the glycine cleavage system genes, resulting in the accumulation of glycine in the body (Table 1). Mutations in the *GLDC* gene are responsible for approximately 80% of NKH cases, while variants in the AMT gene account for roughly 20% [4]. Notably, pathogenic variants of the *DLD* gene have not yet been demonstrated to affect glycine levels in affected individuals. Biallelic variants in *GCSH* gene have recently been reported to cause NKH [5,6].

The accumulation of glycine predominantly impacts the central nervous system. The majority of patients exhibit neurodevelopmental delays, drug-resistant epilepsy, and severe encephalopathy that progresses rapidly. These clinical manifestations, particularly the seizures, are thought to be caused by the overstimulation of the NMDA receptors as glycine is an activator of NMDA receptors in neurons [43]. Current therapeutic strategies focus on the inhibition of NMDA receptors utilizing NMDA antagonists such as ketamine or dextromethorphan, alongside pharmacological interventions aimed at reducing blood glycine levels through the administration of sodium benzoate [44]. Additionally, the implementation of a ketogenic diet has been shown to decrease glycine levels in individuals with NKH [45]. The underlying mechanism by which the ketogenic diet lowers the glycine pool is likely attributable to the utilization of glycine for gluconeogenesis, induced by carbohydrate restriction, rather than the state of ketosis itself [46].

### 2.2. Glutamate Degradation

**Figure 1:**
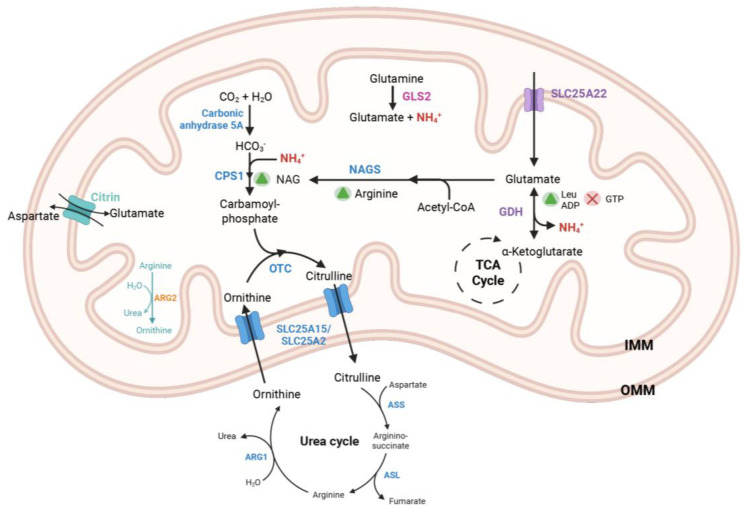
Metabolic pathway of urea cycle in the liver mitochondria. The sources of ammonia in the mitochondria include free ammonia and ammonia generated through the catabolism of glutamine and glutamate via the enzymes GLS2 and GDH, respectively. Glutamate is transported into the mitochondria through the mitochondrial carriers SLC25A22 and Citrin. GDH is activated by ADP and leucine, while it is inhibited by GTP. The α-ketoglutarate produced from the GDH reaction enters the TCA cycle. CPS1 catalyzes the formation of carbamoyl phosphate from bicarbonate and ammonia, a reaction that is activated by NAG. NAG is synthesized by the enzyme NAGS and is regulated by the amino acid arginine. OTC facilitates the conversion of ornithine and carbamoyl phosphate into citrulline. Additionally, the SLC25A15 (or its paralog SLC25A2) transporter (ORNT1) exchanges citrulline and ornithine to maintain the continuity of the urea cycle. Final reaction of urea cycle is catalyzed by ARG1 hydrolyzing arginine into ornithine and urea. OMM: Outer mitochondrial membrane; IMM: Inner mitochondrial membrane; NAG: N-acetylglutamate; NAGS: N-acetylglutamate Synthase; Leu: Leucine; ADP: Adenosine diphosphate; GDH: Glutamate Dehydrogenase, GTP: Guanosine triphosphate; TCA: tricarboxylic acid; CPS1: Carbamoyl Phosphate Synthetase 1; OTC: Ornithine transcarbamylase; GDH: Glutamate Dehydrogenase, GLS2: Glutaminase 2, ARG1: Arginase 1; ARG2: Arginase 2; ASS: Argininosuccinate synthase; ASL: Argininosuccinate lyase.

In hepatocytes, glutamate undergoes oxidative deamination within the mitochondrial matrix by the activity of glutamate dehydrogenase (GDH). The enzymatic activity of GDH is crucial for both carbon and nitrogen metabolism, facilitating the conversion of glutamate to ammonium ions (NH_4_^+^) and α-ketoglutarate, utilizing either NAD^+^ or NADP^+^ as cofactors. Notably, GDH is subject to allosteric inhibition by guanosine triphosphate (GTP) and is activated by adenosine diphosphate (ADP) or leucine (Figure 1). Gain-of-function mutations in the *GLUD1* gene, which encodes the GDH protein, are associated with Hyperinsulinism-Hyperammonemia Syndrome (HHS), a disorder that follows an autosomal dominant inheritance pattern (Table 1). De novo mutations are significantly more prevalent than inherited variants, accounting for approximately 80% of all cases [47]. These activating mutations in the *GLUD1* gene disrupt the allosteric binding sites for GTP, rendering GDH unresponsive to GTP-mediated inhibition.

GDH is predominantly expressed in the liver, kidney, brain, and pancreatic β-cells [48]. HHS typically manifests in the neonatal period with symptomatic postprandial hypoglycemia following protein-rich meals, as well as fasting hypoglycemia. In pancreatic β-cells, the resultant α-ketoglutarate enters TCA cycle, subsequently promoting insulin secretion through the inhibition of ATP-sensitive potassium channels. Elevated ammonia levels observed in affected individuals are attributable to impaired synthesis of N-acetylglutamate (NAG), an essential allosteric activator of the urea cycle, stemming from reduced hepatic glutamate pools [49]. Clinical observations indicate that hyperammonemia in these patients is responsive to the administration of N-carbamylglutamate (NCG), an analogue of NAG, thereby supporting the hypothesis that elevated ammonia levels in HHS are a consequence of mitochondrial glutamate depletion [50]. Additionally, patients with HHS are susceptible to atypical absence seizures even in a euglycemic state [51]. It has been suggested that the neurological manifestations of HHS arise from a complex interplay between hyperactive GDH, recurrent episodes of hyperammonemia, and hypoglycemia resulting in an abnormal glutamate balance in the CNS [52].

Moreover, mutations in the *SLC25A36* gene have been implicated in cases of HHS in the absence of pathological variants in *GLUD1* (Table 1) [8]. The *SLC25A36* gene encodes the protein Pyrimidine Nucleotide Carrier 2 (PNC2), which is believed to facilitate the transport of pyrimidine nucleotides, as well as guanine nucleotides, across the inner mitochondrial membrane [53]. Dysfunctional PNC2 results in decreased mitochondrial GTP levels, thereby attenuating the inhibitory regulation of GDH and leading to a secondary overactivation of the enzyme.

The management of HHS focuses on mitigating neurological sequelae that may result from untreated hyperammonemia and hypoglycemia. Treatment strategies include the implementation of a protein-restricted diet, particularly limiting the intake of leucine, as well as the use of diazoxides, which function by inhibiting insulin secretion through the opening of ATP-sensitive potassium channels [54]. Furthermore, the management of residual complications, such as hyperammonemia and epilepsy, relies on symptomatic treatment approaches.

### 2.3. Proline Degradation

Proline is a distinctive amino acid characterized by its cyclic aliphatic side chain. It is one of three amino acids derived from glutamate, alongside arginine and glutamine. Biosynthesis and degradation of proline are interconnected through a common intermediate Δ-1-pyrroline-5-carboxylate (P5C), which then forms glutamate-Υ-semialdehyde (GSAL) in a reversible, non-enzymatic reaction. Proline is synthesized from either glutamate or ornithine via the intermediate GSAL/P5C, which is subsequently reduced to proline by P5C reductase (PYCR) in an NAD(P)H-dependent manner. In mitochondria, the degradation of proline involves two steps catalyzed by proline dehydrogenase (PRODH) and P5C dehydrogenase (P5CDh). PRODH and PCYR form a metabolic relationship known as the proline-P5C cycle [55].

The initial step of the proline degradation pathway is catalyzed by proline oxidase (POX), an FAD-containing enzyme located in the mitochondrial inner membrane. POX facilitates the conversion of proline to P5C. A deficiency in *POX*, also referred to as proline dehydrogenase (*PRODH*), results in an inborn error of proline metabolism known as hyperprolinemia type I (HPI) (Table 1). The *PRODH* gene is situated in a crucial region on chromosome 22 (22q11.21). Microdeletions within the 22q11 region are associated with velocardiofacial syndrome, and hyperprolinemia has been observed in patients with such microdeletions [56].

The subsequent step in the proline degradation pathway is catalyzed by P5C dehydrogenase (P5CDh), an NAD-dependent enzyme found in the mitochondrial matrix. A deficiency in P5CDh, which is encoded by the *ALDH4A1* gene, leads to hyperprolinemia type II (HPII) (Table 1). P5CDh recognizes GSAL, not P5C itself and subsequently converts it to glutamate. The impaired activity of P5CDh results in the accumulation of P5C, which, in excess, deactivates pyridoxal phosphate (PLP) [57]. The secondary deficiency of PLP in HPII can lead to seizures; however, supplementation with high-dose pyridoxine has been reported to mitigate seizure activity [13]. Both forms of hyperprolinemia are inherited in an autosomal recessive manner. The clinical manifestations associated with elevated proline levels in the bloodstream remain incompletely understood, although neurodevelopmental delays, epilepsy, encephalopathy, and psychiatric symptoms have been observed in both types. Notably, increased plasma and urinary P5C levels serve to differentiate HPII from HPI.

### 2.4. Lysine Degradation

**Figure 2:**
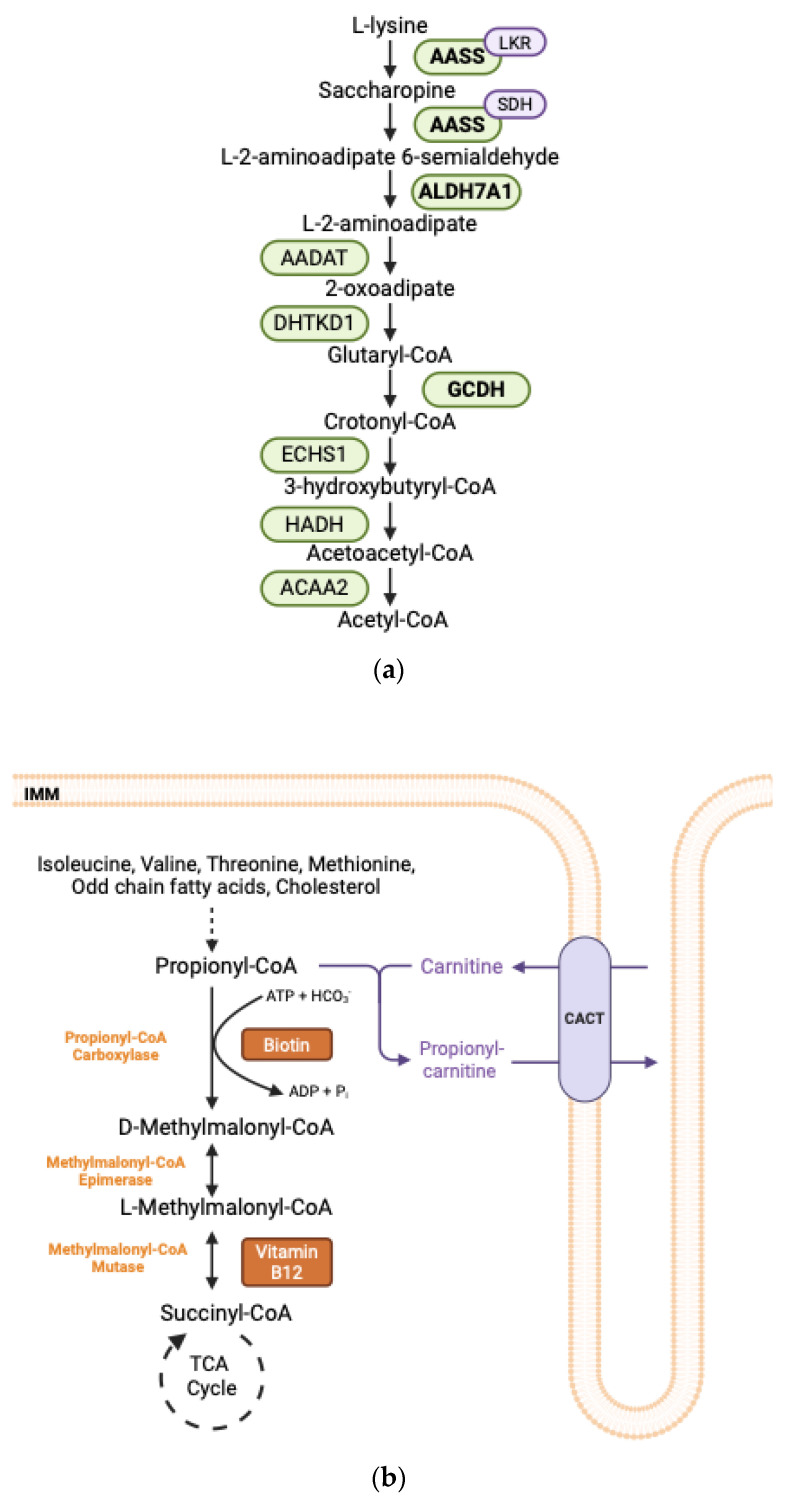
(**a**) Lysine degradation pathway. Enzymes in bold are implicated in human disease. AASS: Alpha-Aminoadipate Semialdehyde Synthase; LKR: Lysine Alpha-Ketoglutarate Reductase; SDH: Saccharopine Dehydrogenase; ALDH7A1: Aldehyde Dehydrogenase Enzyme 7A1; AADAT: Aminoadipate Aminotransferase; DHTKD1: Dehydrogenase E1 and Transketolase Domains-Containing Protein 1; GCDH: Glutaryl-CoA Dehydrogenase; ECHS1: Enoyl-CoA Hydratase, Short-Chain, 1; HADH: Hydroxyacyl-CoA Dehydrogenase, AACA2: Acetyl-CoA Acyltransferase 2. (**b**) Oxidation pathway of propionyl-CoA. Propionyl-CoA is generated through the catabolism of specific amino acids, including isoleucine, valine, threonine, and methionine, as well as from the oxidation of odd-chain fatty acid cholesterol side chains. The first step in the metabolism of propionyl-CoA involves its carboxylation to form D-methylmalonyl-CoA, a reaction catalyzed by PCC which requires biotin as a cofactor. Subsequently, D-methylmalonyl-CoA is isomerized to L-methylmalonyl-CoA, and then L-methylmalonyl-CoA undergoes a rearrangement to yield succinyl-CoA, which can enter the TCA cycle. This rearrangement is catalyzed by methylmalonyl-CoA mutase and necessitates vitamin B12 as a cofactor. In cases of excess propionyl-CoA, propionyl-CoA may be esterified to carnitine, facilitating its transport out of the mitochondria via the carnitine shuttle. The CACT protein mediates the export of propionylcarnitine while concurrently importing free carnitine. ADP: Adenosine diphosphate; ATP: Adenosine triphosphate, Pi: inorganic phosphate, TCA: tricarboxylic acid; CACT: Carnitine-Acylcarnitine Translocase.

Lysine is an essential ketogenic amino acid. Its primary degradation occurs via the saccharopine pathway within liver mitochondria. This metabolic pathway involves nine distinct reactions catalyzed by eight different enzymes. The initial two steps are mediated by a bifunctional enzyme known as 2-aminoadipic acid semialdehyde synthase (AASS). The lysine-ketoglutarate reductase (LKR) domain of AASS catalyzes the conversion of lysine and α-ketoglutarate into saccharopine. Subsequently, the saccharopine dehydrogenase (SDH) domain oxidizes saccharopine to yield α-aminoadipate semialdehyde and glutamate (Figure 2a).

Hyperlysinemia is an autosomal recessive disorder resulting from defects in the *AASS* gene. Isolated mutations in the LKR domain or combined mutations of the LKR and SDH domains lead to hyperlysinemia type I (Table 1). Hyperlysinemia type II is characterized by elevated plasma lysine concentrations and increased urinary excretion of saccharopine, resulting from an isolated deficiency of the SDH domain. To date, hyperlysinemia is generally considered a benign condition that typically does not necessitate treatment [58].

The second enzyme in this degradation pathway, α-aminoadipate semialdehyde dehydrogenase, encoded by the *ALDH7A1* gene, catalyzes the oxidation of α-aminoadipate semialdehyde (α-AASA) to α-aminoadipate (Figure 2a). Deficiencies in *ALDH7A1* are associated with pyridoxine-dependent epilepsy (PDE-ALDH7A1), an autosomal recessive disorder characterized by intractable seizures that respond to pyridoxine treatment (Table 1) [15]. Furthermore, dysfunction in *ALDH7A1* can result in abnormalities in neurological development. Clinical symptoms are attributed to the accumulation of various metabolites, including α-AASA, Δ1-piperideine-6-carboxylate (Δ1-P6C), and pipecolic acid. Notably, Δ1-P6C, the cyclic form of α-AASA, can deactivate pyridoxal-5′-phosphate (PLP), the biologically active form of pyridoxine, leading to seizure activity [59].

The fifth enzyme involved in lysine degradation is glutaryl-CoA dehydrogenase, encoded by the *GCDH* gene. This enzyme catalyzes the oxidative decarboxylation of glutaryl-CoA to crotonyl-CoA (Figure 2a). Deficiency of *GCDH* results in a type of organic acidemia known as glutaric aciduria type I (GA-1) (Table 1). This deficiency leads to the accumulation of neurotoxic metabolites, including glutaric acid and 3-hydroxyglutaric acid. Barzi et al., demonstrated that CRISPR-mediated deletion of the *Aass* gene in the liver halted the production of toxic intermediates in a GA-1 mouse model. Additionally, the rescue of the *Gcdh* gene via liver-directed adeno-associated virus delivery improved neurological phenotypes and increased survival rates. These findings suggest that neurotoxic metabolites originate in the liver and subsequently cross the blood–brain barrier [60].

### 2.5. Branched-Chain Amino Acid Degradation

The oxidation of branched-chain amino acids (BCAAs)—specifically leucine, isoleucine, and valine—serves as a significant energy source for extrahepatic tissues including the brain, kidneys, and skeletal muscle. The degradation pathways of these amino acids involve two critical enzymes: branched-chain amino transferase (BCAT) and the branched-chain α-keto acid dehydrogenase complex (BCKDC). Notably, hepatocytes are deficient in BCAT, rendering the liver incapable of converting BCAAs into their corresponding α-keto acids. However, further processing of α-keto acids occurs in hepatocytes via the activity of BCKDC. BCKDC is a mitochondrial enzyme assembly that requires five distinct cofactors for its activity. The genes *BCKDHA*, *BCKDHB*, *DBT*, and *DLD* encode the respective E1α, E1β, E2, and E3 subunits of this complex. Pathogenic biallelic variants in the *BCKDHA*, *BCKDHB*, or *DBT* genes are responsible for the inborn error of metabolism known as Maple Syrup Urine Disease (MSUD) (Table 1). This autosomal recessive disorder is characterized by the accumulation of BCAAs and their corresponding α-keto acids within the organism. Additionally, variants in the *PPM1K* gene, which encodes an enzyme that activates the BCKDH complex through dephosphorylation, have been implicated in a milder form of MSUD [25].

For the catabolism of leucine, BCKDH catalyzes the conversion of α-ketoisocaproate into isovaleryl-CoA, a process followed by a series of reactions that are unique to leucine degradation. Specifically, isovaleryl-CoA is converted into 3-methylcrotonyl-CoA by isovaleric acid-CoA dehydrogenase, a flavoenzyme encoded by the *IVD* gene. Isovaleric acidemia, an autosomal recessive disorder, results from biallelic pathogenic variants in the *IVD* gene (Table 1). ‘Sweaty feet’ odor is a characteristic feature of isovaleric acid accumulation. Metabolic decompensations during the neonatal period often present with nonspecific neurological symptoms, including vomiting, lethargy, seizures, and failure to thrive. Laboratory findings include metabolic acidosis with anion gap, hyperammonemia due to N-acetylglutamate synthase (NAGS) inhibition, elevated plasma levels of isovalerylcarnitine, and increased urinary excretion of isovalerylglycine [61].

The subsequent enzymatic reaction converting 3-methylcrotonyl-CoA to 3-methylglutaconyl-CoA is facilitated by biotin-dependent 3-methylcrotonyl-CoA carboxylase (MCC). The α- and β-subunits of this enzyme are encoded by the *MCCC1* and *MCCC2* genes, respectively. Deficiencies in either gene lead to 3-methylcrotonyl-CoA carboxylase deficiency (3-MCCD) (Table 1). Although 3-MCCD is frequently detected in newborn screening, it has been reported that most patients diagnosed through newborn screening remain asymptomatic with normal developmental outcomes [62]. The clinical manifestations of 3-MCCD can vary significantly, with excessive accumulation of leucine potentially exerting neurotoxic effects. Neurological toxicity may present as hypotonia, seizures, and developmental delays in affected individuals. Dietary interventions, including L-carnitine supplementation and protein restriction, are considered effective strategies for symptom management.

### 2.6. Degradation of Isoleucine, Valine, Threonine, Methionine

The degradation pathways of isoleucine, valine, threonine, and methionine, as well as odd-chain fatty acids and cholesterol, converge at the formation of propionic acid. Propionic acid, or propionyl-CoA, undergoes carboxylation by the enzyme propionyl-CoA carboxylase (PCC) to produce methylmalonyl-CoA, which is subsequently converted into succinyl-CoA by methylmalonyl-CoA mutase. Succinyl-CoA is a pivotal intermediate that contributes to the TCA cycle and gluconeogenesis (Figure 2b). Propionic acidemia and methylmalonic acidemia are autosomal recessive inborn errors of metabolism resulting from dysfunctional propionyl-CoA carboxylase and methylmalonyl-CoA mutase, respectively.

Propionic acidemia is characterized by mutations in the *PCCA* and/or *PCCB* genes, which encode the α- and/or β-subunits of PCC, respectively (Table 1). Biotin serves as an essential cofactor for PCC; thus, its dysfunction leads to the accumulation of propionyl-CoA within the body. The age of onset varies among patients, with the neonatal period being the most frequently observed. Metabolic decompensation may present with hyperammonemia, metabolic acidosis with an elevated anion gap, abnormal liver function tests, and neurological symptoms such as hypotonia and vomiting. It has been posited that the inhibitory effect of propionyl-CoA on NAGS enzyme contributes to the pathophysiology of hyperammonemia [63]. During episodes of metabolic decompensation accompanied by hyperammonemia, significant reductions in plasma levels of glutamine [64] and alanine [65] have been documented. Long-term management strategies involve the prevention of hyperammonemia through the administration of NCG, dietary restriction of propiogenic amino acids, and the use of antibiotics to reduce propionate levels derived from gut microbiota. Additionally, the incorporation of carnitine into the diet facilitates the excretion of propionic acid by forming propionylcarnitine (Figure 2b) [66].

Isolated methylmalonic acidemias (MMA) encompass a spectrum of organic acidemias caused by impaired activity of methylmalonyl-CoA mutase, which is encoded by the *MUT* gene and follows an autosomal recessive inheritance pattern. The MUT enzyme catalyzes the conversion of methylmalonyl-CoA to succinyl-CoA, utilizing adenosylcobalamin (AdoCbl) as a cofactor (Figure 2b). Genetic defects in either the *MUT* gene or those involved in the adenosylcobalamin synthetic pathway, specifically *MMAA* (cblA) or *MMAB* (cblB) gene defects, primarily account for MMA. Complete absence of MUT activity is classified as mut0, while residual low-to-moderate activity in the presence of elevated AdoCbl levels is designated as partial (mut-). Deficiencies in other cytosolic enzymes within the cobalamin metabolic pathway, such as cblC, cblD, cblF, and cblJ, disrupt the folate cycle, leading to the accumulation of homocysteine and a reduction in methionine production. This group of inborn errors of cobalamin metabolism is collectively referred to as combined methylmalonic acidemia and homocystinuria [67]. The clinical manifestations and therapeutic approaches for MMA closely parallel those for propionic acidemia. Forms of MMA responsive to vitamin B12 (primarily cblA-type) exhibit more favorable clinical outcomes when diagnosed promptly [68].

## 3. Urea Cycle and Ammonia Detoxification

The catabolism of amino acids leads to the generation of nitrogenous waste products, prominently including ammonia, which is a highly toxic by-product of protein metabolism. In mammals, toxic ammonia is converted into water-soluble urea for excretion. This metabolic process is known as the urea cycle, comprising a series of enzymatic reactions that occur exclusively within hepatocytes. The initial substrate for the urea cycle is free ammonia, which is delivered to the hepatic mitochondria primarily in the form of glutamate or glutamine (Figure 1). Glutamate is regarded as the principal amino acid reservoir for amino groups. Final step of the urea cycle is catalyzed by Arginase 1 (ARG1) in hepatocytes, converting arginine into urea and ornithine. Its paralog, Arginase 2 (ARG2), is expressed in macrophages and has been shown to regulate mitochondrial dynamics and bioenergetics [69]. Hepatic Arg2, on the other hand, is upregulated upon fasting and sufficient to enhance basal thermogenesis [70]. Extrahepatic tissues typically synthesize glutamine, which is subsequently transported to the liver for the extraction of ammonia from its amide group.

Disruptions in urea cycle enzymes or transporters result in hyperammonemia and a spectrum of urea cycle disorders (UCDs). These often present with vomiting, lethargy, seizures, and encephalopathy, especially during catabolic stress or protein overload. Hyperammonemia is the hallmark symptom of proximal (mitochondrial) UCDs and requires immediate therapeutic interventions, including the use of nitrogen scavengers, the cessation of dietary protein intake, and, in severe cases, dialysis to remove excess ammonia.

### 3.1. CPS1 Deficiency

Carbamoyl phosphate synthetase 1 (CPS1) catalyzes the initial and rate-limiting step of the urea cycle. This enzymatic reaction involves the incorporation of ammonia, bicarbonate, and two molecules of ATP to produce carbamoyl phosphate. N-acetylglutamate (NAG) acts as an allosteric activator of CPS1, enhancing its enzymatic activity (Figure 1).

Deficiency of CPS1 (CPS1D) is classified as a rare autosomal recessive disorder, with clinical manifestations that closely resemble those of other proximal urea cycle disorders (Table 1). The impaired formation of carbamoyl phosphate leads to decreased urinary excretion of orotic acid. To date, over 270 distinct mutations in the *CPS1* gene have been identified, which are distributed across all exons with the exception of exon 6 [30].

### 3.2. OTC Deficiency

Ornithine transcarbamoylase (OTC) catalyzes the final mitochondrial reaction of the urea cycle, facilitating the incorporation of carbamoyl phosphate into ornithine to form citrulline (Figure 1). This enzyme is predominantly expressed in the liver and small intestine. OTC deficiency (OTCD) is recognized as the most prevalent urea cycle disorder. Due to its X-linked recessive inheritance pattern, the disorder primarily affects male hemizygous mutation carriers (Table 1). Over 500 distinct pathogenic mutations in the *OTC* gene have been documented to date [71]. The severity of clinical symptoms varies significantly, largely depending on residual enzymatic activity. Complete absence of OTC activity is commonly observed in hemizygous males, resulting in severe hyperammonemia during the neonatal period. OTCD has been extensively studied in the context of potential gene therapy interventions due to its relative commonality, well-characterized monogenic basis, and the availability of preclinical models [72].

### 3.3. NAGS Deficiency

N-acetylglutamate synthase (NAGS) catalyzes the synthesis of N-acetylglutamate (NAG), which serves as an essential allosteric activator of CPS1. L-arginine functions as an activator for the NAGS enzyme (Figure 1). Excess organic acids, such as methylmalonyl-CoA and propionyl-CoA, compete with acetyl-CoA in the NAGS-catalyzed reaction, leading to enzymatic inhibition [63].

NAGS deficiency (NAGSD) is one of the rarest urea cycle disorders, with an estimated incidence of 1 in 3,500,000 to 7,000,000 individuals [73]. Clinical presentation typically involves hyperammonemia during infancy (Table 1). Mutations affecting the binding of L-arginine (e.g., the Glu360Asp variant) and variants in noncoding regulatory regions of the NAGS gene have been shown to impair NAGS activity, thereby resulting in the NAGSD phenotype [74]. Treatment for NAGS deficiency includes the administration of carglumic acid (N-carbamylglutamate, or NCG), a bioavailable analog of NAG. NCG bypasses the NAGS reaction and activates CPS1. In cases of unexplained hyperammonemia, the administration of NCG has been suggested as a diagnostic test to assess responsiveness in ammonia levels [75].

### 3.4. Carbonic Anhydrase Va (CA-VA) Deficiency

Carbonic anhydrases (CAs) are enzymes that catalyze the formation of bicarbonate through the hydration of carbon dioxide (Figure 1). CA-Va and CA-Vb are isozymes located within the mitochondria. Hepatic mitochondria utilize bicarbonate in four distinct enzymatic reactions: carbamoyl phosphate synthetase 1, pyruvate carboxylase, propionyl-CoA carboxylase, and 3-methylcrotonyl-CoA carboxylase. CA-VA deficiency has been identified as a potential cause of episodic hyperammonemia (Table 1) [34]. In conjunction with elevated ammonia levels, lactic acidemia and ketonuria are typically observed during metabolic decompensation episodes. Symptoms may present later in life compared to other urea cycle disorders. The deficiency of bicarbonate for the CPS1 reaction contributes to the proximal urea cycle disorder phenotype. Interestingly, NCG has been shown to be beneficial in the treatment of hyperammonemia in patients with CA-VA deficiency [76]. Although the exact mechanism remains to be elucidated, it is hypothesized that HCO_3_^−^ deficiency impairs propionyl-CoA carboxylase activity leading to propionyl-CoA accumulation. Elevated propionyl-CoA competes with acetyl-CoA, inhibiting NAGS and thereby reducing NAG production. The concurrent reduction in NAG and HCO_3_^−^ compromises CPS1 activity, resulting in a secondary hyperammonemia. Therefore, NCG in CA-VA deficiency can potentially restore CPS1 function by bypassing the NAG deficiency [35].

## 4. Mitochondrial Transporters in Amino Acid Metabolism

### 4.1. Hyperornithinemia-Hyperammonemia-Homocitrullinuria Syndrome (SLC25A15 Deficiency)

Hyperornithinemia-hyperammonemia-homocitrullinuria (HHH) syndrome is a rare autosomal recessive urea cycle disorder resulting from a deficiency in the *SLC25A15* gene, which encodes ornithine carrier 1 (ORC1). ORC1 plays a critical role in facilitating the transport of ornithine into the mitochondria and citrulline out of the mitochondria, thus enabling proper urea cycle function (Figure 1). Dysfunction in ORC1 results in the accumulation of ornithine in the cytosol, leading to hyperornithinemia. The consequent reduction of mitochondrial ornithine levels diminishes the activity of OTC, resulting in elevated carbamoyl phosphate levels and subsequent hyperammonemia. Increased carbamoyl phosphate levels, due to decreased OTC activity, further account for the increased urinary excretion of orotic acid as carbamoyl phosphate enters the pyrimidine synthesis pathway. Clinical manifestations of HHH syndrome are akin to those observed in other proximal urea cycle disorders (Table 1). Affected individuals experience symptoms of hyperammonemia, including lethargy, vomiting, failure to thrive, seizures, and, in severe cases, coma [36].

### 4.2. Citrin (SLC25A13) Deficiency

Citrin, also known as the aspartate-glutamate carrier isoform 2, is an inner mitochondrial membrane transporter encoded by the *SLC25A13* gene. Citrin functions as an antiporter, facilitating the export of aspartate from and the import of glutamate into the mitochondrial matrix (Figure 1). In the absence of functional Citrin, insufficient aspartate levels in the cytosol lead to impaired NADH oxidation, causing disturbances in multiple metabolic pathways, including the urea cycle, glycolysis, fatty acid synthesis, gluconeogenesis, and galactose metabolism [77].

Clinical manifestations of Citrin deficiency are contingent upon the age at presentation, with neonatal intrahepatic cholestasis due to Citrin deficiency (NICCD) occurring in the neonatal period, and later presenting as failure to thrive and dyslipidemia (FTTDCD) in childhood, alongside adult-onset type II citrullinemia (CTLN2) (Table 1) [78]. Patients with Citrin deficiency typically exhibit a marked preference for protein-rich and/or lipid-rich foods, accompanied by aversion to carbohydrate-rich foods.

Treatment strategies are tailored to the age at which symptoms present. For NICCD, dietary management involving fat-soluble vitamins, alongside lactose-free and medium-chain triglyceride (MCT)-enriched formulas, is essential. For FTTDCD, the administration of sodium pyruvate may promote growth in conjunction with dietary modifications [79]. The management of CTLN2 is more complex, often necessitating liver transplantation to mitigate metabolic decompensations. Dietary supplementation with arginine to lower blood ammonia levels, alongside sodium pyruvate and MCT oil, has proven beneficial in managing symptoms until transplantation can be performed [80].

### 4.3. SLC25A22 Deficiency

SLC25A22 (Glutamate Carrier 1) functions as an inner mitochondrial symporter that imports glutamate and protons (H^+^) into the mitochondrial matrix (Figure 1). While glutamate transport via SLC25A22 predominantly occurs in the brain, it is also considered an important transporter in the liver, particularly in the fed state [81]. Within the hepatic context, imported glutamate enters the glutamate dehydrogenase (GDH) reaction, yielding ammonia and 2-oxoglutarate. SLC25A22 is abundantly expressed in astrocytes; however, dysfunction of the SLC25A22 protein results in increased extracellular glutamate levels, activating extrasynaptic glutamate receptors. Pathogenic variants in *SLC25A22* have been associated with early infantile epileptic encephalopathy (EIEE) type 3 and epilepsy of infancy with migrating focal seizures (EIMFS) (Table 1). EIEE type 3 is characterized by myoclonic seizures, hypotonia, microcephaly, and a suppression-burst pattern on electroencephalography. The pathophysiological mechanisms underlying these conditions may be attributed to the accumulation of glutamate in astrocytes [82].

## 5. Mitochondrial Amino Acid Metabolism and Cancer

Metabolic reprogramming is one of the hallmarks of cancer [83]. The metabolism of amino acids plays a crucial role not only in supporting the survival of cancer cells through protein synthesis and energy production, but also in facilitating various redox reactions and epigenetic modifications via diverse mechanisms [84,85]. The epigenetic regulation is closely linked to one-carbon metabolism through protein and nucleic acid methylation [86]. Methionine is converted by methionine adenosyltransferase (MAT) into S-adenosylmethionine (SAM), the main methyl donor for reactions modifying lysine and arginine residues on proteins, as well as DNA, RNA, and metabolites. SAM is then demethylated to S-adenosylhomocysteine (SAH). Methylation-driven silencing of tumor suppressor genes, dependent on SAM levels, is a key early step in oncogenesis for many cancers [87]. Additionally, glutathione (GSH), the most abundant antioxidant in living organisms, plays a vital role in maintaining cellular redox homeostasis. It is a tripeptide composed of glutamic acid, cysteine, and glycine. GSH exhibits a dual role in cancer progression [88]. A decrease in GSH levels is associated with increased susceptibility to oxidative stress, which plays a critical role in cancer progression. Conversely, elevated intracellular GSH concentrations enhance the cellular antioxidant capacity and confer resistance to oxidative damage, a phenomenon frequently observed in various malignant cells [89].

Glutamate dehydrogenase 1 (GLUD1) exhibits dichotomous effects across different cancer types. Specifically, *GLUD1* is overexpressed in certain malignancies, such as non-small cell lung cancer (NSCLC) [90]; while it is downregulated in hepatocellular carcinoma (HCC) and clear cell renal carcinoma [91]. Zhao et al., demonstrated that *GLUD1* overexpression inhibits the proliferation of HCC cells and tumor growth both in vitro and in vivo, whereas *GLUD1* knockdown promotes HCC progression [92]. Additionally, GLUD1 is considered an important druggable target. Epigallocatechin gallate (EGCG), an inhibitor of GLUD1 and GLUD2, has been shown to suppress the proliferation of *IDH1*-mutated cancer cell lines in vitro [93]. Another GLUD1 inhibitor, R162, yielded promising results by effectively decreasing cellular proliferation in patient-derived xenograft mouse models of NSCLC [90].

A variety of cancer types utilize glutamine as a primary energy source to sustain their proliferative capacity, primarily through enhanced glutamine uptake and glutaminolysis [94,95]. The liver-type glutaminase (encoded by *GLS2*) can exhibit dualistic effects on tumorigenesis depending on tumor type. Saha et al., utilized the Oncomine database to reveal that *GLS2* is significantly overexpressed in bladder, colon, rectal, head-and-neck, peritoneal, and lung cancers; while its expression is notably decreased in brain, liver, and pancreatic cancers [96]. Elucidating the tumor-suppressive mechanisms of GLS2 in the context of liver cancer, Suzuki et al., demonstrated that GLS2 can inhibit HCC in vivo by promoting ferroptosis. This process is facilitated by the increased production of lipid reactive oxygen species (ROS) resulting from the conversion of glutamate to α-ketoglutarate [95].

Another significant aspect of metabolic reprogramming in cancer is the role of carbamoyl phosphate (CP). As previously noted, CP represents the first product of the rate-limiting step in the urea cycle. Concurrently, CP is synthesized in the cytosol by a multi-domain enzyme known as CAD, which utilizes glutamine, carbon dioxide, and ATP as substrates in the de novo pyrimidine biosynthesis pathway. Cancer cells can exploit mitochondrial pools of CP generated by the CPS1 enzyme for de novo pyrimidine biosynthesis. This occurs through the overexpression of *CPS1*, leading to elevated CP levels in the mitochondria, which are subsequently translocated to the cytosol. This mechanism was demonstrated in a subset of NSCLC, where the concurrent activation of oncogenic KRAS and the loss of liver kinase B1 (LKB1) promoted tumor growth [97]. Furthermore, *CPS1* overexpression is associated with poor prognostic outcomes in cholangiocarcinoma [98]. In contrast, *CPS1* expression is often downregulated through hypermethylation in HCC, where low CPS1 levels correlate with elevated CAD expression, potentially favoring the channeling of glutamine towards the CAD reaction to initiate the de novo pyrimidine synthesis pathway [99].

Similar to *CPS1* overexpression, reduced levels of OTC can lead to increased CP accumulation in the mitochondria due to decreased consumption of CP as a substrate. Decreasing OTC expression facilitates the diversion of ornithine into alternative metabolic pathways, including polyamine biosynthesis, which promotes the survival of cancer cells [100]. Notably, reduced OTC expression has been reported in patient samples of HCC, suggesting a potential mechanism by which cancer cells may adapt to their metabolic environment [101].

## 6. Conclusions and Perspectives

Mitochondrial amino acid metabolism in the liver supports diverse physiological functions, from nitrogen disposal and energy production to redox balance and biosynthesis. These pathways are subcellularly compartmentalized, tightly regulated, and responsive to nutrient availability. Disruptions—whether genetic or acquired—can trigger systemic toxicity, often with disproportionate effects on the central nervous system. Many inborn errors of metabolism are rooted in defects in mitochondrial enzymes or transporters and are characterized by toxic metabolite accumulation, hyperammonemia, and episodic decompensation.

Currently, the primary objectives of therapeutic interventions include the removal of toxic byproducts, supplementation of essential amino acids or cofactors, dietary modifications to inhibit the formation of toxic metabolites, and limiting the availability of intermediates to decelerate disease progression. Rapid increase in the level of toxic metabolites due to various mechanisms including infections and medications can precipitate metabolic decompensation. Decompensated patients require vigilant monitoring and prompt initiation of symptomatic treatment. However, management of these patients presents considerable challenges in the face of poor prognostic factors such as high number of metabolic decompensations, poor patient compliance with interventions, and further clinical deterioration. The ideal treatment in poorly controlled metabolic decompensation is liver transplantation. Transplantation restores the function of the deficient enzyme. Urea cycle disorders and organic acidemias account for a significant proportion of pediatric liver transplant cases [102]. Liver transplantation has been shown to improve clinical outcomes [103]. However, the limited availability of donor organs poses a significant barrier to achieving optimal outcomes for many patients. Additionally, postoperative complications and the effects of prolonged immunosuppression underscore the urgent need for novel therapeutic strategies.

Emerging technologies are beginning to transform the landscape. Gene therapy approaches—such as adeno-associated virus (AAV)–mediated gene addition and mRNA delivery—are under clinical investigation for disorders like propionic acidemia and OTC deficiency. These strategies aim to restore enzyme function directly in hepatocytes and offer the potential for long-term correction. For instance, a Phase I/II open-label dose-optimization study of mRNA-3927 is actively recruiting participants (NCT04159103). mRNA-3927 is a lipid nanoparticle (LNP)-encapsulated dual mRNA therapy that encodes for PCCA and PCCB subunits of PCC in the liver aiming to achieve sufficient enzyme levels [104]. Additionally, a Phase III randomized, double-blind, placebo-controlled trial of DTX301 is being conducted in patients with late-onset ornithine transcarbamylase (OTC) deficiency (NCT05345171). DTX301 utilizes adeno-associated virus vector serotype 8 (scAAV8) to encode human OTC [105]. Musunuru et al., recently reported the development of a personalized lipid nanoparticle-delivered base editing strategy to treat a neonate with CPS1 deficiency [106]. The therapeutic approach employed an adenine base editor (termed kayjayguran abengcemeran or k-abe) to correct the *CPS1* Q335X “stop” variant, a non-sense G-to-A variant on the noncoding strand. Post-treatment reductions in plasma ammonia levels were interpreted as evidence of a successful correction of the pathogenic CPS1 variant. While long-term follow-up is necessary, this work represents a significant advancement in the application of individualized genomic editing strategies for the treatment of inborn errors of metabolism. Parallel efforts in genome editing and transporter targeting may expand therapeutic possibilities beyond classical enzyme deficiencies.

Moreover, aberrant metabolite levels that are frequently associated with human diseases serve as valuable indicators for elucidating disease mechanisms and identifying biomarkers for diagnosis, prognosis, and therapeutic monitoring. Given the central role of metabolic reactions in physiological processes, the use of metabolomic profiling has enabled the identification of previously unrecognized links between disease states and specific metabolic pathways. Recent large-scale Genome-Wide Association Studies (GWAS) of the human metabolome have uncovered widespread genetic contributions that underlie individual variation in metabolic profiles [107]. Other methods such as The Gene-Metabolite Association Prediction (GeneMAP) platform were developed to identify metabolic gene functions by integrating genetic models of gene expression with gene-mediated regulation of metabolites [108]. These platforms have the potential of advancing our understanding of mechanisms for diseases.

Collectively, advancing our mechanistic understanding of mitochondrial amino acid metabolism in the liver will inform both rare disease management and novel strategies for targeting diseases such as cancer.

## Figures and Tables

**Table 1:** Overview of inborn errors of amino acid metabolism associated with deficiencies of proteins in liver mitochondria.

Amino Acid	Disease	Inheritance	Biochemical Markers	Genes	Notable Reported Mutations
Glycine	Nonketotic hyperglycinemia (MIM#605899)	AR	High serum and CSF glycine levels (high CSF-to-plasma glycine ratio)	*GLDC*	Coughlin et al. identified p.R515S as the most common missense mutation. Other recurrent missense mutations were listed as p.S564I, p.G761R, p.A389V, p.A802V, p.R790W, p.R905G, p.G771R, and p.R461Q [4].
				*GCSH*	Majethia et al. reported a biallelic start loss variant, c.1A > G [5] and Arribas-Carreira et al. reported pathogenic variants including c.1A > G (p.(Met1?)) and c.226C > T (p.(Gln76*)) in patients presenting with combined NKH and lipoate deficiency [6].
				*AMT*	Coughlin et al. reported that p.R320H mutation was the most common missense mutation identified in 16% of all *AMT* alleles [4].
Glutamate	Hyperinsulinism—Hyperammonemia Syndrome (MIM#606762)	AD	Leucine-sensitive hypoglycemia High ammonia levels	*GLUD1*	Luczkowska et al. reported that c.1496G > T (p.(Gly499Val)) variant in *GLUD1* altered the allosteric sensitivity to both inhibitory action of GTP and activation by ADP, rendering cells metabolically responsive to glutamine [7].
				*SLC25A36*	The only reported pathogenic variants are c.284 + 3A > T [8,9] and c.803dupT (p.Ser269llefs*35) [10].
Proline	Hyperprolinemia type I (MIM#239500)	AR	High serum proline levels without urinary P5C excretion	*PRODH*	Hama et al. described a case of a patient with HPI and harboring the c.1397 C > T (p.T466M) mutation [11].
	Hyperprolinemia type II (MIM#239510)	AR	High serum proline levels with high urinary P5C	*ALDH4A1*	Kaur et al. detected a nonsense homozygous variant, c. 1633C > T, in the *ALDH4A1* gene [12]. Motte et al. identified two compound heterozygous variants in an adult patient: c.62 + 1G > A, which is located in intron 1 and results in aberrant splicing, and c.349G > C, a missense variant in exon 5 that affects a highly conserved residue [13].
Lysine	Hyperlysinemia type I (MIM#238700)	AR	High plasma lysine levels	*AASS*	Yeganeh et al. identified biallelic variants in *AASS*, c.436C > T (p.R146W) and c.1112C > T affecting the folding of the lysine-2-oxoglutarate domain of AASS [14].
	Pyridoxine-dependent epilepsy (MIM#266100)	AR	High plasma levels of pipecolic acid, α-aminoadipic semialdehyde (AASA) and its cyclic form Δ1-piperideine-6-carboxylate (P6C)	*ALDH7A1*	More than 165 pathogenic variants have been reported to date [15]. Gene variant c.1279G > C (p.E427Q) was the most frequently reported, followed by c.834G > A (p.V278V) and c.1364T > C (p.L455P) [16].
	Glutaric aciduria type I (MIM#231670)	AR	High levels of glutaric acid (GA), 3-hydroxyglutaric acid (3-OH-GA), glutaconic acid, and glutarylcarnitine (C5DC) in various bodily fluids	*GCDH*	c.1204C > T (p.Arg402Trp) is the most prevalent pathogenic variant in various populations followed by c.1262 C > T (p.Ala421Val) [17,18].
BCAA (Leucine, Isoleucine, Valine)	Isovaleric acidemia (MIM#243500)	AR	High urinary C5-carnitine (C5) metabolites, isovalerylglycine (IVG), and 3-hydroxyisovaleric acid (3-HIVA)	*IVD*	The distribution of hotspots varies significantly across various ethnic backgrounds. c.941C > T (p.Ala314Val) is the most common variant in patients identified through newborn screening in multiple populations [19,20].
	MCC deficiency (MIM#210200)	AR	High urinary excretion levels of 3-hydroxyisovaleric acid (3-HIVA) and 3-methylcrotonylglycine (3-MCG). 3-hydroxyisovalerylcarnitine (C5OH) is high in plasma and urine.	*MCCC1* and *MCCC2*	According to the newborn screening data from a Chinese province, variants with the highest frequency were listed as c.639 + 2T > A, c.1679dupA, c.980C > G in *MCCC1* gene and c.1144-1147delinsTTTT, c.416C > T, c.1073–6T > A, c.735dupC, c.470A > G in *MCCC2* gene [21].
	MSUD type IA (MIM#248600)	AR	High levels of alloisoleucine (chemical derivative of isoleucine) Elevated plasma branched-chain amino acids (isoleucine, leucine, and valine) Elevated urine branched-chain ketoacids	*BCKDHA*	Miragaia et al. presents a case with a patient who developed acute encephalopathy, later revealed a pathogenic variant c.659C > T (p.A220V) in homozygosity in the *BCKDHA* gene [22].
	MSUD type IB (MIM#620698)			*BCKDHB*	Among MSUD patients, pathogenic variants of *BCKDHB* are more commonly seen in most populations. Chen et al. show that c.331C > T in the *BCKDHB* gene is a relatively common variant in MSUD with a frequency of 5.8%. [23].
	MSUD type II (MIM#620699)			*DBT*	c.280C > T, c.433 + 2T > G, c.500del, c.1264dup, c.1268T > C variants in the *DBT* gene are reported to be causing MSUD phenotype [24].
	Variant MSUD (MIM#611065)			*PPM1K*	A homozygous truncating mutation (c.417delTA) [25] and a homozygous stop-loss mutation (c.1A-G) [26] are the only reported mutations in the literature to date.
	Propionic acidemia (MIM#606054)	AR	High plasma propionylcarnitine (C3) and glycine levels High urinary 3-hydroxypropionate excretion in addition to the presence of methylcitrate, tigylglycine, propionylglycine and lactic acid	*PCCA* and *PCCB*	In a Chinese cohort, c.2002G > A and c.937C > T were reported to be the most frequent variants in *PCCA* gene; while c.838dupC was the most common *PCCB* variant, followed by c.1087T > C, c.1228C > T and c.1283C > T in *PCCB* gene [27].
	Methylmalonic acidemia (MIM#251000)	AR	High plasma methylmalonic acid and glycine High urinary methylmalonic acid and methylcitrate excretion	*MMUT*	Yu et al. identified 144 different mutations in *MMUT* gene in 266 Chinese patients. Patients harboring the mutations c.1663G > A, c.2080C > T, c.1880A > G, and c.1208G > A exhibited complete responsiveness to vitamin B12 treatment. In contrast, those with the mutations c.1741C > T, c.1630_1631GG > TA, and c.599T > C demonstrated partial responsiveness to the same treatment [28].
Urea Cycle	CPS1 deficiency (MIM#237300)	AR	Hyperammonemia with low plasma levels of citrulline and arginine and high levels of glutamine Low or normal levels of orotic acid in urine.	*CPS1*	According to HGMD, more than 340 *CPS1* mutations have been reported [29]. It has been illustrated that the extensive genetic heterogeneity at the CPS1 locus, wherein private mutations are the norm, while the recurrence of mutations is observed only as an exception [30]. The only currently known recurrent *CPS1* mutation, c.3037_3039del (p.Val1013del) is found to be a recurrent mutation in the Turkish population [31].
	OTC deficiency (MIM#311250)	XR	Hyperammonemia with low levels of citrulline and arginine, high levels of glutamine and alanine, and normal ornithine levels. High urinary excretion of orotic acid.	*OTC*	Kido et al. evaluated 523 variants in Japanese patients and found a degree of genotype–phenotype correlation in male OTCD patients, with all 53 detected nonsense variants associated with the neonatal-onset phenotype [32].
	NAGS deficiency (MIM#237310)	AR	Hyperammonemia with low levels of citrulline and arginine, high levels of glutamine. Low or normal levels of orotic acid in urine.	*NAGS*	Heibel et al. described the first disease-causing enhancer mutation in *NAGS*. The homozygous mutation, c.-3064C > A, affects a highly conserved nucleotide within the hepatic nuclear factor 1 (HNF-1) binding site, significantly impairing *NAGS* gene expression and thereby causing the NAGSD phenotype in the reported patient [33].
	CA5A deficiency (MIM#615751)	AR	Hyperammonemia and hyperlactatemia with high glutamine and low-to-normal citrulline levels without orotic aciduria	*CA5A*	Only a small number of mutations have been identified. Two clinical missense mutations, c.697T > C (p.Ser233Pro) and c.721G > A (p.Glu241Lys) were shown to decrease enzyme activity in vitro [34,35].
AA Transporters	Hyperornithinemia—hyperammonemia—homocitrullinuria syndrome (MIM#238970)	AR	Hyperammonemia with high plasma ornithine, and high homocitrulline excretion	*SLC25A15*	HHH has been reported in less than 100 cases, with less than 45 variants described. Two common mutations are considered to be p.F188del and p.R179* [36].
	Citrin deficiency (MIM#605814)	AR	Hyperammonemia with high plasma levels of citrulline and arginine. Increased plasma threonine-to-serine ratio.	*SLC25A13*	Specific *SLC25A13* variants such as c.852_855delTATG, c.1177 + 1G > A, IVS16ins3kb, and c.1638_1660dup are notably prevalent within East Asian cohorts [37].
	SLC25A22 deficiency (MIM#609304)	AR	High plasma proline levels with intermittently elevated plasma ornithine or arginine levels.	*SLC25A22*	Only a small number of cases have been reported to date. Some of these mutations include c.835dupG; p.Glu279Gly [38]; c.617C > T; p.Pro206Leu [39]; c.706G > T; p.Gly236Trp [40]; c.328G > C; p.Gly110Arg [41]; c.97A > G; p.Lys33Glu [42].

## Data Availability

No new data were created or analyzed in this study.
